# *Schistosoma japonicum* EKLF/KLF1 is a potential immune target to tackle schistosomiasis

**DOI:** 10.1186/s13071-023-05947-2

**Published:** 2023-09-23

**Authors:** Xianyu Piao, Ning Jiang, Shuai Liu, Jiamei Duan, Hang dai, Nan Hou, Qijun Chen

**Affiliations:** 1 NHC Key Laboratory of Systems Biology of Pathogens, Institute of Pathogen Biology, Chinese Academy of Medical Sciences & Peking Union Medical College, Beijing, China; 2 Key Laboratory of Livestock Infectious Diseases in Northeast China, Ministry of Education, Key Laboratory of Ruminant Infectious Disease Prevention and Control (East), Ministry of Agriculture and Rural Affairs, College of Animal Science and Veterinary Medicine, Shenyang Agricultural University, Shenyang, China; 3 The Research Unit for Pathogenic Mechanisms of Zoonotic Parasites, Chinese Academy of Medical Sciences, Shenyang, China; 4 Institute of Biological Products, National Institutes for Food and Drug Control, Beijing, China

**Keywords:** Diagnosis, EKLF, KLF1, *Schistosoma japonicum*, Schistosomiasis

## Abstract

**Background:**

Interruption of parasite reproduction by targeting migrating schistosomula is a promising strategy for managing schistosomiasis. Hepatic schistosomula proteins previously identified based on second-generation schistosome DNA sequencing were found to hold excellent potential for schistosomiasis japonica diagnosis and as vaccine candidates. However, there are still many unknown schistosomula proteins that warrant further investigations. Herein, a novel schistosomula protein, the *Schistosoma japonicum* erythroid Krüppel-like factor (*Sj*EKLF/KLF1), was explored.

**Methods:**

Sequence alignment was carried out to detect the amino acid sequence characteristics of *Sj*EKLF. The expression profile of *Sj*EKLF was determined by western blot and immunofluorescence analysis. Enzyme-linked immunosorbent assay was used to determine the antigenicity of *Sj*EKLF in hosts. Mice immunised with recombinant *Sj*EKLF were challenged to test the potential value of the protein as an immunoprotective target.

**Results:**

*Sj*EKLF is defined as EKLF/KLF1 for its C-terminal DNA-binding domain. *Sj*EKLF is mainly expressed in hepatic schistosomula and male adults and located within the intestinal intima of the parasites. Notably, high levels of *Sj*EKLF-specific antibodies were detected in host sera and *Sj*EKLF exhibited outstanding sensitivity and specificity for schistosomiasis japonica immunodiagnosis but failed to distinguish between ongoing infection and previous exposure. In addition, *Sj*EKLF immunisation reduced the infection in vivo, resulting in decreased worm and egg counts, and alleviated body weight loss and hepatomegaly in infected mice.

**Conclusions:**

Overall, these findings demonstrate that *Sj*EKLF is critical for the infection of *S. japonicum* and may be a potential target to help control *S. japonicum* infection and transmission.

**Graphical Abstract:**

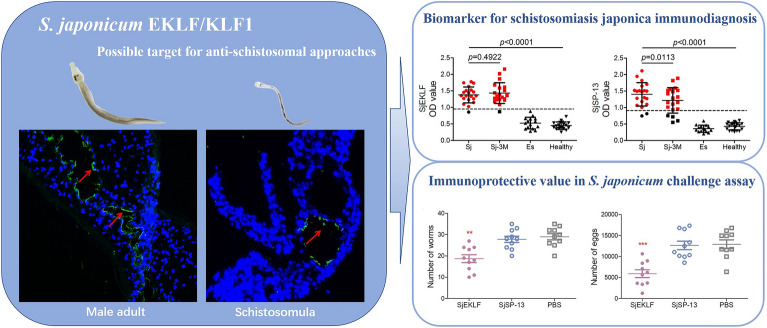

**Supplementary Information:**

The online version contains supplementary material available at 10.1186/s13071-023-05947-2.

## Background

The prevalence of schistosomiasis has not diminished substantially over the past 2 to 3 decades [1]. Results from an online survey of 365 experts on neglected tropical diseases suggested that current approaches focused primarily on mass drug administration may not be effective to eliminate schistosomiasis [2]. Compared with schistosomiasis haematobium and schistosomiasis mansoni, the endemic region of schistosomiasis japonica is decreasing, primarily in Asian countries such as the Philippines and China [3, 4]. However, it is still necessary to recognize disease spreading patterns due to natural disasters and/or climate change. Thus, the development of improved diagnostic methods and efficacious vaccines is very important for preventing and controlling schistosomiasis.

Schistosomes have complex life cycles. In the host, schistosomula migrate from the infection site in the skin epidermis and travel along the blood vessels and then through the lungs. Schistosomula arrive at the hepatic portal system at approximately 14 days post infection (dpi), settle down and undergo sexual maturation. Eggs laid by female worms lead to the major pathologies associated with schistosomiasis [5, 6]. Migrating schistosomula are in direct contact with the host circulation and are directly exposed to the host humoral immune response; therefore, they are the main targets of schistosomiasis drugs and vaccines to prevent infection.

In a previous study, based on second-generation schistosome DNA chip data, we identified a set of genes whose expression was highest in the hepatic schistosomula, the early stage of *Schistosoma japonicum*, compared with that in cercariae, male adults, female adults and eggs [7]. A group of hepatic schistosomula proteins encoded by these genes was successfully generated, and five exhibited excellent qualities as schistosomiasis japonica diagnostic and vaccine candidates. However, more than 60 proteins encoded by the identified genes have not been successfully prepared and investigated. In particular, the hepatic schistosomula transcriptional profile of a putative gene (GenBank ID: CNUS0000098854.1) was found to encode the *S. japonicum* KLF1 protein (*Sj*EKLF, GenBank ID: TNN19877.1) by sequence alignment.

Krüppel-like factors (KLFs) belong to a family of transcription factors that comprise 18 proteins in humans [8–10], which are important constituents of the eukaryotic transcriptional machinery and regulate the expression of a wide variety of genes. The first mammalian KLF was identified in red blood cells and therefore was named erythroid Krüppel-like factor (EKLF) [11]. EKLF, which is also known as Krüppel-like factor 1 (KLF1), consists of two domains: a N-terminal proline-rich transactivation domain and a C-terminal highly conserved DNA-binding domain comprising three C2H2 zinc fingers that recognise the consensus-binding site [12–14]. EKLF plays a multifunctional and essential role at virtually all stages of erythrocyte development [15–18]. Indeed, recent studies revealed that EKLF promotes the growth, migration and invasion of cancer cells [19–21].

To date, most studies on EKLF were performed in placental mammals and some in model organisms, such as zebrafish, but KLFs in pathogens have not been reported. *Sj*EKLF was herein investigated further. The characteristics of this protein and its antigenicity during schistosome infection were explored in the present study.

## Methods

### Animals and parasites

Snails infected with *S. japonicum* (*Oncomelania hupensis*) were provided by the Jiangxi Provincial Institute of Parasitic Diseases, Jiangxi, China. Freshly released cercariae stimulated by light were immediately harvested. Six-week-old pathogen-free male BALB/c mice and New Zealand white rabbits (both from Vital River Laboratory Animal Technology Co., Beijing, China) were percutaneously infected with cercariae (40 ± 2 per mouse and 1000 ± 100 per rabbit). Serum samples were collected from the infected animals at 0, 7, 14, 21, 28, 35, 42 and 56 dpi. Hepatic schistosomula (14 dpi) and adult worms (42 dpi) were manually isolated by portal perfusion under a light microscope via the vascular system of the infected mice. Eggs were purified from the liver tissues of infected mice at 42 dpi by enzyme digestion, as previously described [22].

### Human samples

Patients with schistosomiasis japonica (*n* = 20), echinococcosis (*n* = 15) and healthy volunteers (*n* = 20) were recruited in Hunan Province, Xinjiang Uyghur Autonomous Region and Heilongjiang (a province non-endemic for schistosomiasis japonica), China, from March 2016 to February 2017. Sera were collected from all participants at enrolment and again 3 months after praziquantel treatment in the case of schistosomiasis patients. All schistosomiasis cases were confirmed by examining EPG (eggs per gram of faeces) using the Kato-Katz method. Only three patients were moderately infected (EPG > 120), and the rest were mildly infected. After 3 months of praziquantel treatment, EPG was 0 in all patients except one case with 2. Cystic echinococcosis was confirmed by clinical diagnosis, parasitological detection and medical history records. Patient information is presented in Table 1.

**Table 1:** Clinical characteristics of the enrolled subjects whose sera were used in ELISA for diagnosis

Subjects	Schistosomiasis patients n = 20	Echinococcosis patients n = 15	Healthy individuals n = 20
Age mean^*^ (years)	51.25 ± 8.29	53.87 ± 10.99	50.0 ± 13.02
Age range (years)	33–62	33–70	28–70
Male/female	19/1	14/1	18/2
EPG^#^ mean*	46 ± 71.01		
EPG^#^ range	3–220		
Province	Hunan	Xinjiang	Heilongjiang
Race	Chinese	Chinese	Chinese

^*^Median ± SD

^#^Eggs per gram of stool (assessed by Kato-katz technique)

### Sequence analysis

The amino acid sequence of EKLFs from *Schisotosoma japonicum* (TNN19877.1), *S. mansoni* (XP_018648096.1), *Homo sapiens* (NP_006554.1), *Mus musculus* (NP_034765.3), *Rattus norvegicus* (NP_001100634.1) and *Bos taurus* (NP_001073828.1) were obtained from GenBank. Conserved domains of these proteins were analysed using Conserved Domain Search Service [23]. The sequences were aligned using DNAMAN V9.0 (https://www.lynnon.com).

### Quantitative real-time polymerase chain reaction (qRT-PCR)

QRT-PCR was performed as previously described [24]. Briefly, total RNA from parasites were extracted using RNeasy Mini Kit (Qiagen, Hilden, Germany) and reverse-transcribed into cDNA using the Invitrogen SuperScript III reverse transcriptase kit (Thermo Fisher Scientific, Waltham, MA, USA) according to the manufacturer’s instructions. The 26S proteasome non-ATPase regulatory subunit 4 (*PSMD4*; GenBank ID: FN320595) was used as reference gene [25]. Reactions detecting the expression of glyceraldehyde-3-phosphate dehydrogenase (*GAPDH*, GenBank ID: FN324551) in standard cDNA (equally mixed cDNA from parasites at each developmental stage) were used as standard controls. The primers for the genes were designed using Primer BLAST (https://www.ncbi.nlm.nih.gov/tools/primer-blast/) and were the following: *SjEKLF*: forward primer, 5′-GAGTCACACATCCGAACTGA-3′ and reverse primer, 5′-ACATGTGCAACCTGACAACTG-3′; *PSMD4*: 5′-CCTCACCAACAATTTCCACATCT-3′, 5′-GATCACTTATAGCCTTGCGAACAT-3′; *GAPDH*:5′-ATGGAACAAGGATGGTGCTGAG-3′; 5′-CAACAAACATGGGTGCGTCT-3′. QRT-PCR was performed in technical triplicates using Brilliant II SYBR Green QPCR Master Mix Kit (Agilent Technologies, Santa Clara, CA, USA) and an Applied Biosystems 7500 Real-time PCR System (Thermo Fisher Scientific), according to the manufacturers’ instructions. The data were analysed using Applied Biosystems 7500 system software version 1.3.1, and relative copy numbers were computed according to the 2^−ΔΔCt^ method using a statistical confidence interval of 95%. Gene expression values within parasites of specified developmental stages were normalised to the standard control (relative expression value = (relative copy number of *Sj*EKLF in parasites of specific stage/relative copy number of GAPDH in the standard parasite cDNA) × 100%).

### Recombinant protein production and polyclonal antibody generation

The *Sj*EKLF-encoding gene was cloned using Gateway Technology with Clonase II (Invitrogen, Thermo Fisher Scientific, Waltham, MA; USA) according to the manufacturer’s instructions, as previously described [26]. Primers were designed using Primer BLAST (forward primer: 5′-GGGGACAAGTTTGTACAAAAAAGCAGGCTTCATGTCACACATTTGGAATGCTAGA-3′; reverse primer: 5′-GGGGACCACTTTGTACAAGAAAGCTGGGTCCTAGGTATGAATTCGATAATGAGCC-3′). His-tagged fusion proteins were purified using Ni–NTA Agarose (Qiagen, Hilden, Germany), according to the manufacturer’s instructions. The molecular weights of the recombinant and natural proteins were predicted using ProtParam (https://web.expasy.org/protparam/). The selected gene fragments were amplified from schistosome cDNA using high-fidelity Phusion DNA polymerase (Finnzymes Oy, Finland). The amplified product was purified using DNA Gel Extraction Kit (Axygen, CA, USA) and then cloned into the entry plasmid pDONR^TM^221 by the BP recombination reaction. Entry plasmids were then used to perform the LR recombination reaction to transfer the gene fragments into expression plasmid pDEST^TM^17. Positive clones in *Escherichia coli* Transetta (DE3) (TransGen Biotech, Beijing, China) were selected for sequencing to obtain the expression plasmids with the correct reading frame. The fusion proteins with His-tags were purified with Ni-NTA Agarose (QIAGEN) according to the manufacturer’s instruction. Proteins were analysed by western blotting using monoclonal antibodies against His-tag (Cell Signalling Technology, Danvers, MA, USA). Recombinant *S. japonicum* secreted protein 13 (*Sj*SP-13) was generated as previously described [27]. Rabbit polyclonal antibodies were prepared by Beijing Protein Innovation (Beijing, China) by immunising New Zealand white rabbits with the recombinant proteins.

### In vitro cultivation of parasites

Mice were infected with male cercariae to generate hepatic schistosomulum (200 ± 20 per mouse) and male adults (40 ± 2 per mouse). Hepatic schistosomulum and male adults were obtained at 14 dpi and 42 dpi respectively. Then, schistosomulum (100 per well) and adults (20 per well) were cultured in 24-well plates in RPMI 1640 for 8 h. The supernatants were collected and concentrated using Amicon Ultra-15 Centrifugal Filters (Ultracel-10K, Merck Millipore, Carrigtwohill, IRL). These concentrations were then used for western blot.

### Western blot

Parasites were homogenised and incubated with lysis buffer (8 M urea, 4% CHAPS, 1% dithiothreitol, 1% EDTA, 10 mM Tris and 35 μg/ml phenylmethylsulfonyl fluoride). Protein concentration was quantified using a BCA kit (Pierce Biotechnology, Waltham, MA, USA) in accordance with the manufacturer’s instructions. Recombinant or extracted proteins were separated on 12% sodium dodecyl-sulfate polyacrylamide gel electrophoresis (SDS-PAGE) gels and transferred onto polyvinylidene difluoride membranes (Millipore, Bedford, MA, USA). Sera from *S. japonicum*-infected humans, mice and rabbits (diluted 1:500) with sera from uninfected individuals and animals as negative controls, rabbit polyclonal antibodies against recombinant *Sj*EKLF (r*Sj*EKLF; diluted 1:1000) and anti-actin antibody produced in rabbit affinity isolated antibody (Sigma-Aldrich, St. Louis, MO, USA) with rabbit IgG as a control were used as primary antibodies. IRDye 800 CW conjugated goat anti-human IgG (H + L) antibodies, goat anti-mouse IgG (H + L) antibodies and goat anti-rabbit IgG (H + L) antibodies (all from Li-COR Biosciences, Lincoln, NE, USA) were used for the final incubation. Detection was performed using an Odyssey system (Li-COR Biosciences).

### Enzyme-linked immunosorbent assay (ELISA)

ELISA was performed as previously described [7]. Briefly, plates (96 well) were coated with 1 μg/ml r*Sj*EKLF overnight at 4 °C. Human IgG, mouse IgG and rabbit IgG were used as positive controls. Serum samples (diluted 1:100) were added to the wells. Goat anti-mouse, anti-rabbit and anti-human polyvalent immunoglobulin (α-, γ-, and μ-chain specific) conjugated to alkaline phosphatase (Sigma-Aldrich) were used as secondary antibodies (1:10,000). Optical density (OD) values were measured at 405 nm. The OD values on different plates were weighted by the OD value of the control IgG at 0.1 μg/ml. The cutoff value of the positive test was set at 2.1 times the mean OD value of serum samples from healthy individuals [28]. Sensitivity was defined as true positives/(true positives + false negatives) and specificity as true negatives/(false positives + true negatives).

### Immunofluorescence

Immunofluorescence was performed to detect the localisation of *Sj*EKLF. Serial cryosections (5–7 μm) were obtained and fixed in 4% formaldehyde. Total IgG from the sera of rabbits immunised with r*Sj*EKLF was purified using a Protein A Sepharose Fast Flow Kit (GE Healthcare, Chicago, IL, USA) according to the manufacturer’s instructions. The sections were blocked with 5% bovine serum albumin buffer (in phosphate-buffered saline [PBS]) for 2 h at 25 °C and then incubated with rabbit polyclonal antibodies against r*Sj*EKLF (2 mg/ml, 1:500 dilution) and control IgG in blocking solution overnight at 4 °C. Cells were detected using Alexa Fluor 555 donkey anti-rabbit IgG (H + L) and 4′,6-diamidino-2-phenylindole (DAPI, all from Invitrogen) and observed using a TCS SP5 confocal microscope (Leica Microsystems, Wetzlar, Germany).

### Immunization and challenge experiments

*Sj*EKLF-immunised and control groups (10 mice per group) were first immunised with 60 µg r*Sj*EKLF or an equal volume of PBS emulsified with complete Freund’s adjuvant by subcutaneous injection and then with 30 µg protein or PBS emulsified with incomplete Freund’s adjuvant, respectively, every 2 weeks for a total of three immunisations. After immunisation, the mice were infected with cercariae (40 ± 2 parasites per mouse) released by infected *O. hupensis*. Adult worms and eggs were isolated and counted at 42 dpi. The experiment was repeated twice.

### Statistical analysis

Data were analysed using Prism 5.0 (GraphPad, San Diego, CA, USA) and Microsoft Excel 2010 (Microsoft, Redmond, WA, USA). The statistical significance of the experimental data was evaluated between two groups using two-tailed paired Student’s *t*-test or two-tailed Mann-Whitney test, and among more groups using one-way analysis of variance. Statistical significance was set at *P* < 0.05.

## Results

### Characteristics of *Sj*EKLF coding sequence

**Fig. 1:**
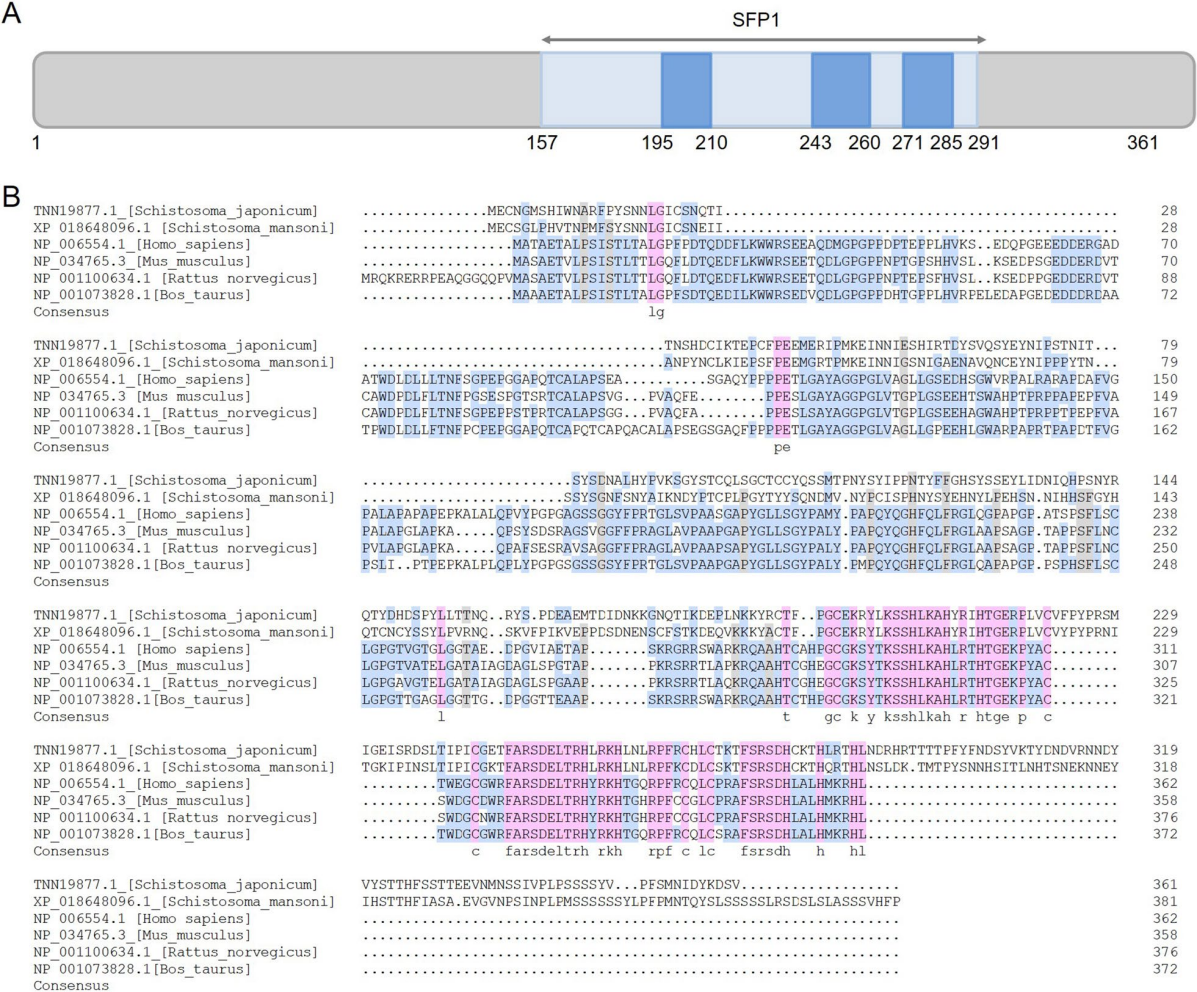
Sequence analysis of *Schistosoma japonicum* erythroid Krüppel-like factor (*Sj*EKLF). **a** Schematic representation of the SjEKLF (TNN19877.1) amino acid sequence. The dark blue block indicates the domain of SFP1 super family and the light blue blocks indicate C2H2 zinc fingers. **b** The sequences of EKLFs from *Schisotosoma japonicum*, *S. mansoni*, *H. sapiens*, *M. musculus*, *R. norvegicus* and *B. taurus* were obtained and aligned. Pink indicates homology 100%, grey indicates homology > 75% and blue indicates homology > 50%

Sequence alignment revealed that *Sj*EKLF has a C-terminal highly conserved DNA-binding domain comprising three C2H2 zinc fingers (amino acid, aa 157–361, Fig. 1A, B). *Sj*EKLF had approximately just 16% homology with the EKLFs from *H. sapiens* (NP_006554.1), *M. musculus* (NP_034765.3), *R. norvegicus* (NP_001100634.1) and *B. taurus* (NP_001073828.1) EKLF proteins (Fig. 1B). The proline content in the N-terminal of *Sj*EKLF (aa 1–156) is only 7.1%, while the proline contents in the N-terminal domain of EKLFs from *H. sapiens* (aa 10–279), *M. musculus* (aa 10–275), *R. norvegicus* (aa 28–293) and *B. taurus* (aa 10–289) are 14.8%, 15.8%, 16.2% and 17.5%, respectively (Fig. 1B).

### Protein of *Sj*EKLF is mainly expressed in hepatic schistosomula and male adults

**Fig. 2:**
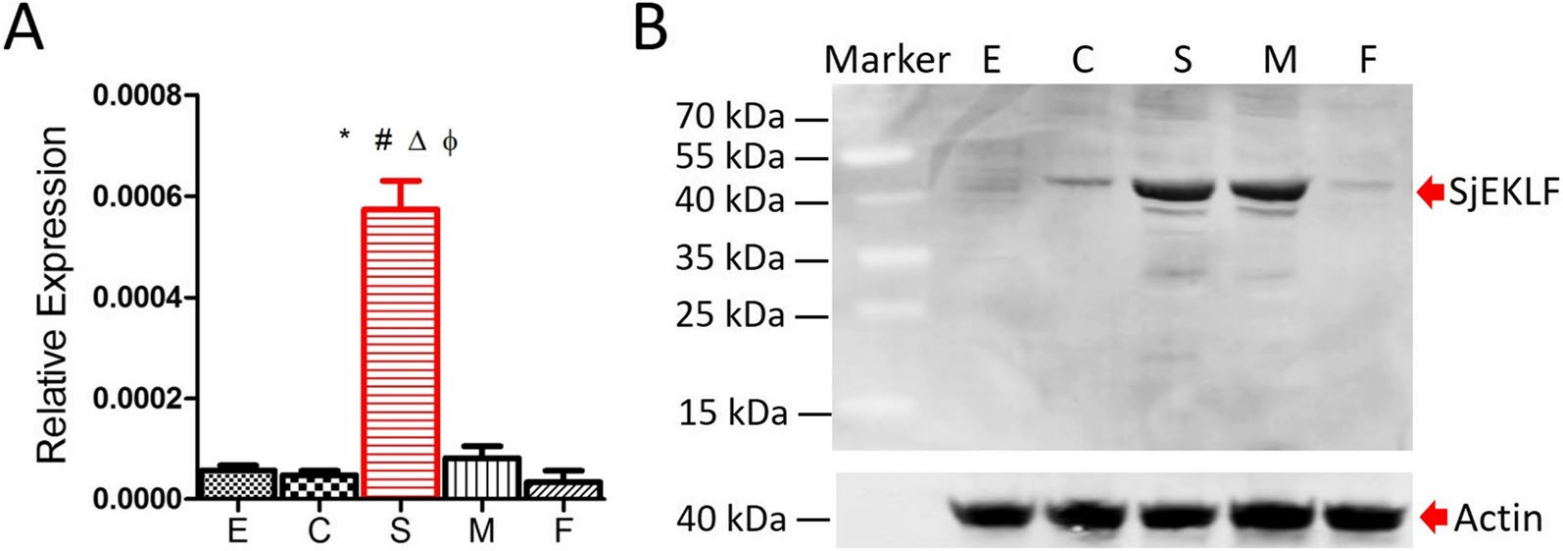
Expression of *Schistosoma japonicum* erythroid Krüppel-like factor (*Sj*EKLF) in different developmental stages of the parasite. **a** Transcriptional expression of the *Sj*EKLF-encoding gene in the five developmental stages of *S. japonicum* detected by quantitative real-time polymerase chain reaction. Data are shown as mean + SD. *, #, Δ and Φ indicate comparison with the E, C, M and F groups, respectively. *, #, Δ and Φ indicate* P* < 0.01 by Mann-Whitney test. **b** Expression of *Sj*EKLF and actin in the five developmental stages of *S. japonicum* were detected by western blot with 20 µg protein lysate uploaded per lane for *Sj*EKLF detection and 5 µg for actin detection. Red arrows indicate the target protein bands. Abbreviations: E, egg; C, cercariae; S, hepatic schistosomula; M, adult male; F, adult female

**Fig. 3:**
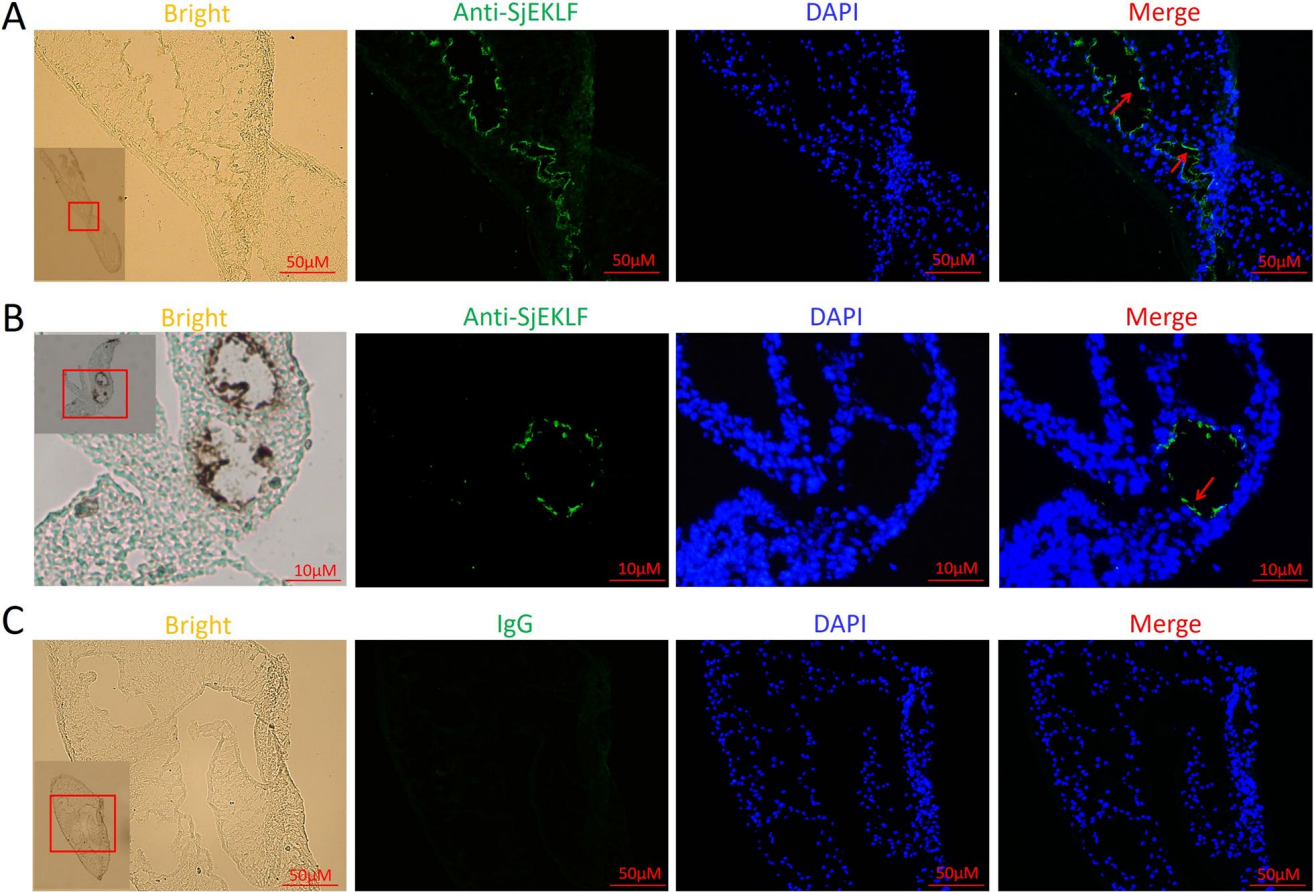
Location of *Schistosoma japonicum* erythroid Krüppel-like factor (*Sj*EKLF) detected by immunofluorescence. Cryosections of male adult (**a**) and hepatic schistosomulum (**b**) samples obtained from *S. japonicum*-infected BALB/c mice were incubated with rabbit anti-SjEKLF polyclonal antibodies; cryosections of male adults were incubated with rabbit IgG as control (**c**) and then with Alex Flour 555 donkey anti-rabbit IgG (green fluorescence). DAPI (blue) was used to stain the nucleus. Red arrows indicate positive staining. These results are representative of three independent experiments

Analysis of the transcriptional expression of *Sj*EKLF revealed that the protein was highly transcriptionally expressed in the hepatic schistosomula compared with the other four stages of the parasite (cercariae, male adults, female adults and eggs, Fig. 2A). Notably, the C-terminal of *Sj*EKLF was highly conserved, in particular the amino acid positions 194–289 that encoded three Krüppel-like C2H2 zinc fingers (Fig. 1). To ensure the specificity of the detection of polyclonal antibodies, gene fragments of *Sj*EKLF encoding the protein fragment comprising the 6–214 amino acids were amplified to construct the clones. A recombinant protein (approximately 30 kDa) of *Sj*EKLF was obtained and further verified by western blotting (Additional file 1: Fig. S1A). Western blotting showed that the natural *Sj*EKLF was approximately 40 kDa (Fig. 2B), which is smaller than the mammal EKLF, but similar to that predicted by the ProtParam tool. *Sj*EKLF was found to be mainly expressed in the hepatic schistosomula and male adults of *S. japonicum* with very low expression in cercariae, female adults and eggs (Fig. 2B). *Sj*EKLF present in the culture supernatant of both hepatic schistosomulum and male adult in vitro (Additional file 1: Fig. S1B). Moreover, immunofluorescence analysis showed that *Sj*EKLF localized within the intestinal intima of the hepatic schistosomula and male adults (Fig. 3).

### *Sj*EKLF exhibits promising qualities for schistosomiasis japonica immunodiagnosis

**Fig. 4:**
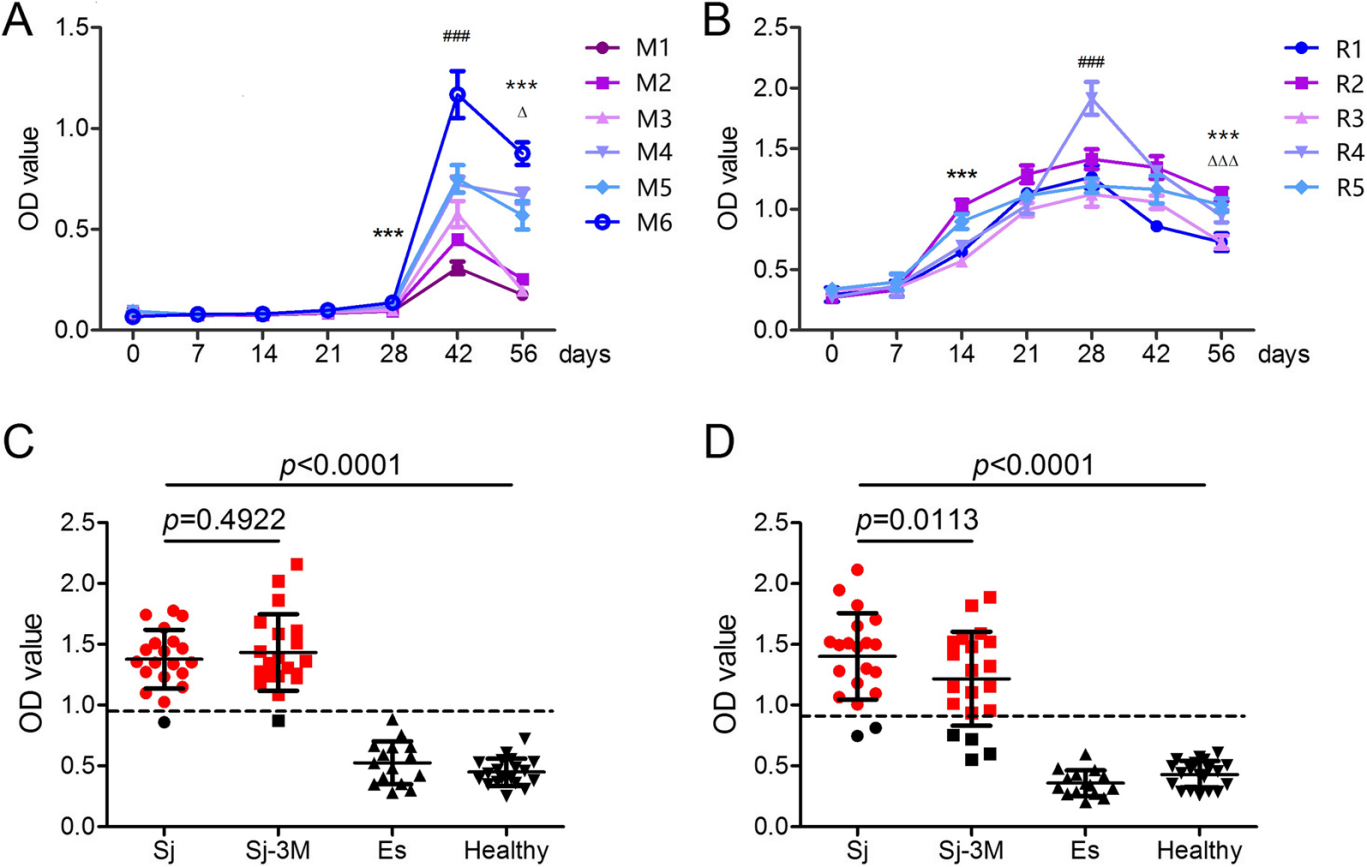
Determination of *Schistosoma japonicum* erythroid Krüppel-like factor (*Sj*EKLF)-specific antibodies in host serum by ELISA. **a** BABL/c mice and **b** New Zealand white rabbits were percutaneously infected with cercariae (40 ± 2 parasites per mouse and 1000 ± 100 parasites per rabbit). Sera from infected animals were collected at 0, 7, 14, 21, 28, 35, 42 and 56 days post infection (dpi). Antibody titres against *Sj*EKLF in these sera were determined by ELISA with1 μg/ml r*Sj*EKLF. Linear charts show the detailed dynamics of antibodies against *Sj*EKLF. *, # and Δ indicate comparisons at 0, 28 and 42 dpi, respectively. Δ indicates *P* < 0.05; ***, ### and ΔΔΔ indicate *P* < 0.0001. M, mouse; R, rabbit. **c** *Sj*EKLF and **d** *Sj*SP-13-specific antibodies in human serum samples were also determined by ELISA. Serum samples from patients with confirmed schistosomiasis japonica before (Sj) and 3 months after praziquantel treatment (Sj-3 M) (*n* = 20 each group), with confirmed echinococcosis (Es) (*n* = 15) and healthy individuals (healthy) (*n* = 20) were included in the assays. Cutoff value of positive tests was set at ≥ 2.1 times the mean optical density (OD) value of healthy individuals (dotted lines). Red and black dots indicate a positive and negative individual, respectively

Sera from *S. japonicum*-infected patients, BALB/c mice and New Zealand rabbits at 42 dpi specifically recognised r*Sj*EKLF (Additional file 1: Fig. S1A). Assessment of the dynamics of *Sj*EKLF-specific IgG in mouse and rabbit sera was carried out by ELISA. The results revealed that the antibody titres in the serum peaked at 42 and 28 dpi in *S. japonicum*-infected mice (Fig. 4A) and rabbits (Fig. 4B), respectively, and then declined. However, the SjEKLF antibody levels at 56 dpi were still significantly higher than those at 0 dpi. Antibodies against *Sj*EKLF in human sera were also detected using ELISA. Serum samples collected from 20 *S. japonicum*-infected patients before and 3 months after praziquantel treatment, 15 echinococcosis patients and 20 healthy individuals were evaluated. Sera from healthy volunteers were used as negative controls, whereas sera from patients with echinococcosis were used to assess test specificity and cross-reactivity. r*Sj*EKLF exhibited outstanding sensitivity (95% [95% confidence intervals, CI: 73.1–99.7%], two-tailed Student’s t-test) and specificity (100% [95% CI: 80.0–100%]) (Fig. 4C). *Sj*SP-13 was recently identified as a novel protein with excellent sensitivity and specificity for the diagnosis of schistosomiasis japonica [28] and was herein used as a control. The results of *Sj*SP-13 (sensitivity: 90% [95% CI: 66.9–98.2%] and specificity: 100% [95% CI: 80.0–100%], two-tailed Student’s t-test) (Fig. 4D) in this study were close to the results (90.4% sensitivity and 98.9% specificity) reported by Xu et al. [28]. Comparison of matching serum samples before and after drug treatment showed that antibody titres against r*Sj*EKLF were not significantly affected.

### *Sj*EKLF immunization can relieve schistosomiasis japonica in mice

**Fig. 5:**
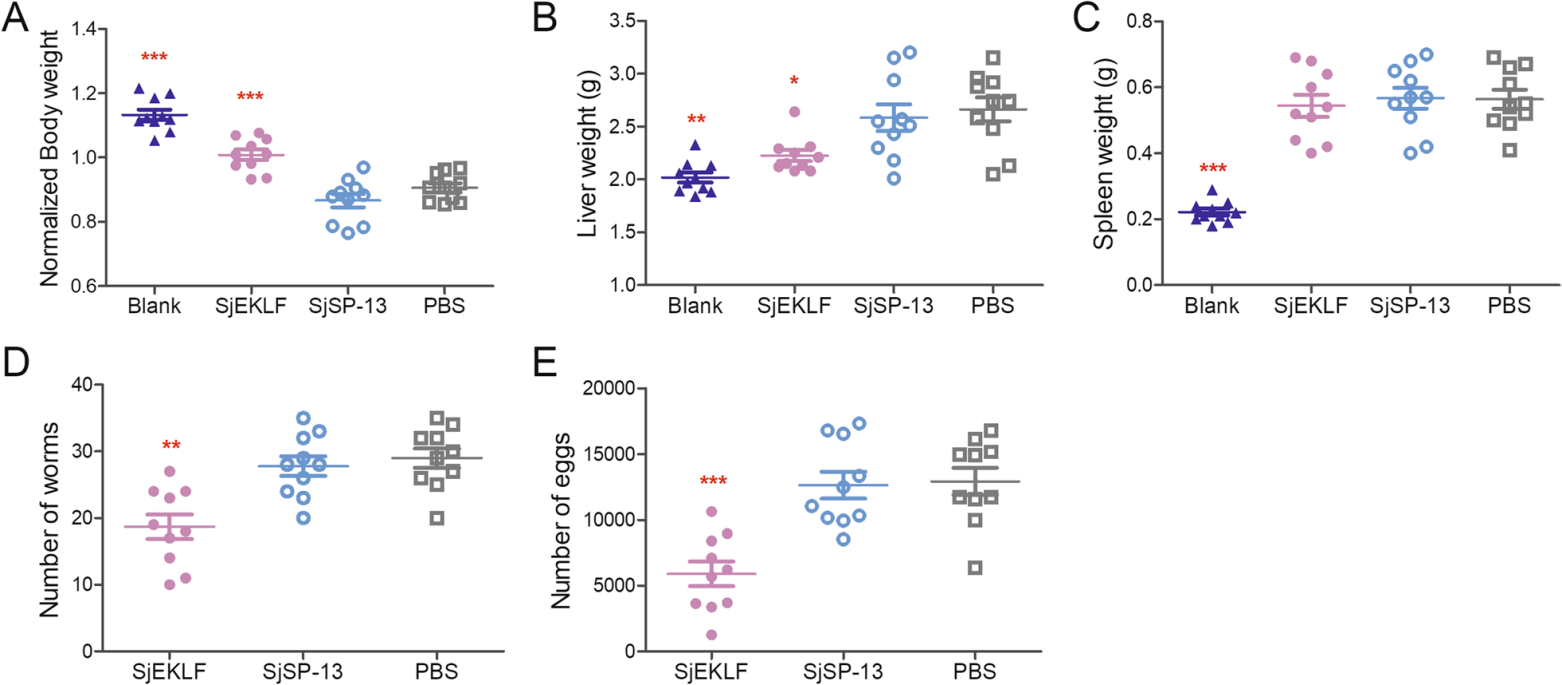
Immunoprotective efficacy of *Schistosoma japonicum* erythroid Krüppel-like factor (*Sj*EKLF) in *S. japonicum*-infected mice. Mice were immunised with His-tagged recombinant *Sj*EKLF, *Sj*SP-13 or PBS (negative control) (*n* = 10 per group). After immunisation, the mice were challenged with cercariae (40 ± 2 per mouse). The Blank group was immunised with PBS and did not receive cercariae. The mice were killed 42 days post infection. **a** Normalized body weight of mice (weight of mice at 42 days post infection/its pre-infection weight), **b** liver weight and **c** spleen weight were determined and compared between the groups. **d** Worms and **f** eggs were collected and counted under a microscope. The results are representative of two independent experiments. *P < 0.05, **P < 0.01, ***P < 0.0001 compared with the PBS group

BALB/c mice were immunised with His-tagged r*Sj*EKLF and were then challenged with *S. japonicum* (40 cercariae per mouse). Overall, the treated animals showed significantly reduced body weight loss (Fig. 5A) and hepatomegaly (Fig. 5B and Additional file 2: Fig. S2) compared with animals non-immunised with r*Sj*EKLF, but this treatment approach had no effect on splenomegaly (Fig. 5C and Additional file 2: Fig. S2). The cercariae used in the challenge experiment were highly infective as the average ratio of adult worms to cercariae in the PBS-immunised group reached 72.5% (Fig. 4D). Immunisation with r*Sj*EKLF significantly reduced the number of worms with the worm reduction rate reaching 34.9% (Fig. 5D, E). In contrast with r*Sj*EKLF immunization, *Sj*SP-13 showed no protective effects (Fig. 5D, E).

## Discussion

The major drug and vaccine strategies for schistosomiasis aim to prevent schistosome infection and/or reduce egg production by interrupting parasite reproduction by targeting migrating schistosomula and adult females [29]. Proteins critical for schistosomula development are promising candidates for schistosomiasis control.

Our previous work revealed the transcriptional profile of hepatic schistosomula [7], which includes a putative gene encoding the *S. japonicum* EKLF protein. EKLF is essential for definitive erythropoiesis [15, 16] and is a critical transcription factor for the expression of the β-globin coding gene in mice and humans [30, 31]. Several studies have elucidated more potential EKLF target genes, including dematin and other components of the red cell cytoskeleton, transcription factors, blood group antigens, heme synthesis enzymes and genes involved in cell cycle regulation [32–34]. Moreover, EKLF is upregulated in cervical and gastric cancer cells, thereby promoting cancer cell proliferation, migration and invasion [20, 35]. EKLF was also reported to promote the proliferation, migration and invasion of human lens epithelial cells [21]. A protein with a high homology degree to EKLF is highly expressed in the epidermal layer of the skin and epithelial cells of the tongue, palate, oesophagus, stomach and colon in mice [36]. *Sj*EKLF was found to locate within the intestinal intima of the parasites, indicating SjEKLF may be involved in the regulation of intestinal endothelial cells.

Herein, we describe the EKLF protein of a pathogen (*S. japonicum*) for the first time to our knowledge. The challenge assay revealed that *Sj*EKLF immunization can relieve schistosomiasis japonica in mice, indicating the important roles of *Sj*EKLF in the parasite infection and development. Specifically, *Sj*EKLF was detected in the culture supernatant of both schistosomula and male adult in vitro and was found to locate in the intestinal lining of the hepatic schistosomula and male adults of *S. japonicum*. ELISA assays showed high levels of *Sj*EKLF-specific antibodies in host sera. These results suggest that *Sj*EKLF may be transported into the schistosome intestinal tract and discharged into the host circulation, thereby triggering a host immune response. Therefore, we hypothesize that SjEKLF may regulate the biological function of intestinal endothelial cells and participate in the digestion and/or absorption of host components and that specific antibodies binding to SjEKLF may block its pathway of action, thus affecting the development of worms. The specific function of *Sj*EKLF in schistosome development still needs further investigation.

Several proteins of *S. japonicum* have been found to have encouraging performance in the immunodiagnosis of schistosomiasis japonica, including *Sj*SP-13 [28], *Sj*SAPLP4, *Sj*SAPLP5 [27], *Sj*ScP80, *Sj*ScP84 and *Sj*ScP88 [7]. The herein described r*Sj*EKLF, a non-conserved fragment of naïve *Sj*EKLF, was also found to exhibit excellent sensitivity (95%) and specificity (100%) for the diagnosis of *S. japonicum*. Furthermore, ELISA assay based on rSjEKLF can effectively identify mildly infected patients, as only three patients included in this study were moderately infected and the rest were mildly infected. In addition, animal experiments showed that the serum titres of *Sj*EKLF antibodies decreased significantly after reaching a peak at 42dpi or 28dpi, while our previous work found *Sj*SP13 antibodies remained high at 56dpi [7]. These results suggest that *Sj*EKLF antibody levels are higher during acute infection at 28–42 dpi but declined when the chronic infection progresses. However, schistosomiasis japonica patients with acute infection were hard to recruit; patients often do not seek medical attention in a timely manner. All patients involved in this study have developed liver lesions and are in a state of chronic infection. Unfortunately, there was no significant difference in serum SjEKLF antibody titres in schistosome patients before and after drug treatment; thus, SjEKLF could not be used to distinguish between ongoing infection and previous exposure.

The highly conserved C-terminal domain of this protein makes it difficult to design drugs that can specifically act on schistosome EKLF without affecting mammalian EKLF activity. In addition to drugs, vaccines represent the most cost-effective method for the long-term control of schistosomiasis. Many candidates from *S. mansoni* and *Schistosoma haematobium* have been subjected to clinical trials, such as *S. haematobium* 28-kD glutathione S-transferase (r*Sh*28GST) [37], *S. mansoni* 14-kDa fatty acid-binding protein (*Sm*14) [38], *S. mansoni* tetraspanin (*Sm*TSP-2) [39] and the large subunit of *S. mansoni* calpain (*Sm*p80) [40]. To date, no candidate for schistosomiasis japonica has been under clinical trials. The important regulatory function of EKLF in cell biology and the high expression of *Sj*EKLF in migrating larvae and hepatic schistosomula make this protein a valuable target for schistosomiasis vaccine design. The sequence outside the N- and C-terminal domains is highly non-conserved, making it possible to design schistosomiasis vaccines targeting *Sj*EKLF. Overall, in vivo immunization with r*Sj*EKLF reduced the number of worms and eggs in *S. japonicum*-infected mice. Schistosomula that are severely affected by *Sj*EKLF antibodies may be eliminated by the host immune system, resulting in fewer worms and eggs in the challenge experiments, while the ones mildly affected may successfully resist the host immune system and eventually mature. Many *S. japonicum* antigens are currently under research, such as triosephosphate isomerase (*Sj*TPI) [41], cytosolic fatty acid-binding protein (*Sj*FABPc) [42], 23-kDa integral membrane protein (*Sj*23) [43] and schistosomulum protein 25 (*Sj*ScP25) [7]. However, the protective effects of these *S. japonicum* antigens in animal studies remain limited, with the highest worm and egg reduction rates reaching only 50% and 65%, respectively, in *S. japonicum*-infected mice. Therefore, research on vaccines for schistosomiasis japonica still requires comprehensive investigations.

In summary, our study revealed that *Sj*EKLF is critical for the infection of *S. japonicum* larvae and exhibits potential value for schistosomiasis japonica immunodiagnosis and immunoprotection. Hence, *Sj*EKLF may pave the way for the development of new strategies to manage *S. japonicum* infection and transmission. However, the specific function of *Sj*EKLF in the development of schistosome still needs further investigation.

### Supplementary Information

**Additional file 1: Fig. S1.** Detection of the naïve and recombinant protein of *Schistosoma japonicum* erythroid Krüppel-like factor (*Sj*EKLF). **a** Recombinant protein of the fragment of *Sj*EKLF (6–214 amino acids) was resolved by 12% odium dodecyl-sulphate polyacrylamide gel electrophoresis and stained with Coomassie brilliant blue staining (Lane 1) and then detected by western blotting with an anti-His-tag mouse monoclonal antibody (Lane 2) or with patient or infected animal sera, including a mixture of serum samples (equal volumes) from 10 schistosomiasis japonica patients (Lane 3) or 10 healthy volunteers (Lane 4), a mixture of serum samples (equal volumes) from six infected BALB/c mice 42 dpi (Lane 5) or normal mice (Lane 6) and a mixture of serum samples (equal volumes) from five infected rabbits 42 dpi (Lane 7) or normal rabbits (Lane 8). **b** Hepatic schistosomulum (100 per well) and male adults (20 per well) were cultured in 24-well plates in RPMI 1640 for 8 h. *Sj*EKLF in the concentrated supernatants of control group (RPMI 1640, lane 1), schistosomula group (lane 2) and male adult group (lane 3) were detected by western blotting with anti-*Sj*EKLF antibodies.

**Additional file 2: Fig. S2.** Effect of *Schistosoma japonicum* erythroid Krüppel-like factor (*Sj*EKLF) immunization on the livers and spleens of *S. japonicum*-infected mice. Mice were immunised with His-tagged recombinant *Sj*EKLF, *Sj*SP-13 (positive control) or PBS (negative control) (*n* = 10 per group). After immunisation, the mice were challenged with cercariae (40 ± 2 per mouse). The PBS group was immunised with PBS and did not receive cercariae. The mice were killed 42 days post infection. The morphology of the livers and spleen from each group of mice is shown. Scale bar indicates 5 mm.

## Data Availability

All data and materials were available in this manuscript.
