# Correction: *Schistosoma japonicum* EKLF/KLF1 is a potential immune target to tackle schistosomiasis

**DOI:** 10.1186/s13071-023-06008-4

**Published:** 2023-10-17

**Authors:** Xianyu Piao, Ning Jiang, Shuai Liu, Jiamei Duan, Hang dai, Nan Hou, Qijun Chen

**Affiliations:** 1 NHC Key Laboratory of Systems Biology of Pathogens, Institute of Pathogen Biology, Chinese Academy of Medical Sciences & Peking Union Medical College, Beijing, China; 2 Key Laboratory of Livestock Infectious Diseases in Northeast China, Ministry of Education, Key Laboratory of Ruminant Infectious Disease Prevention and Control (East), Ministry of Agriculture and Rural Affairs, College of Animal Science and Veterinary Medicine, Shenyang Agricultural University, Shenyang, China; 3 The Research Unit for Pathogenic Mechanisms of Zoonotic Parasites, Chinese Academy of Medical Sciences, Shenyang, China; 4 Institute of Biological Products, National Institutes for Food and Drug Control, Beijing, China

**Correction: Parasites & Vectors (2023) 16:334** 10.1186/s13071-023-05947-2

Following publication of the original article, the following errors were brought to the attention of the journal: the article had been published as a Correspondence article rather than a Research article; the section heading 'Introduction' had been used in place of 'Background'; the section heading 'Materials and Methods' had been used instead of 'Methods'; 'nonparametric Student’s *t*-test' was referred to instead of 'Mann–Whitney test' (which can be seen to be specified in the caption of Fig. 2) in the subsection 'Statistical analysis'. The published article [1] has since been updated to correct these errors.
