# Supplementary figures and images for: Schistosoma japonicum EKLF/KLF1 is a potential immune target to tackle schistosomiasis
Source: Parasit Vectors. 2023 Sep 23;16:334. doi: 10.1186/s13071-023-05947-2 (PMC10517563; doi:10.1186/s13071-023-05947-2)

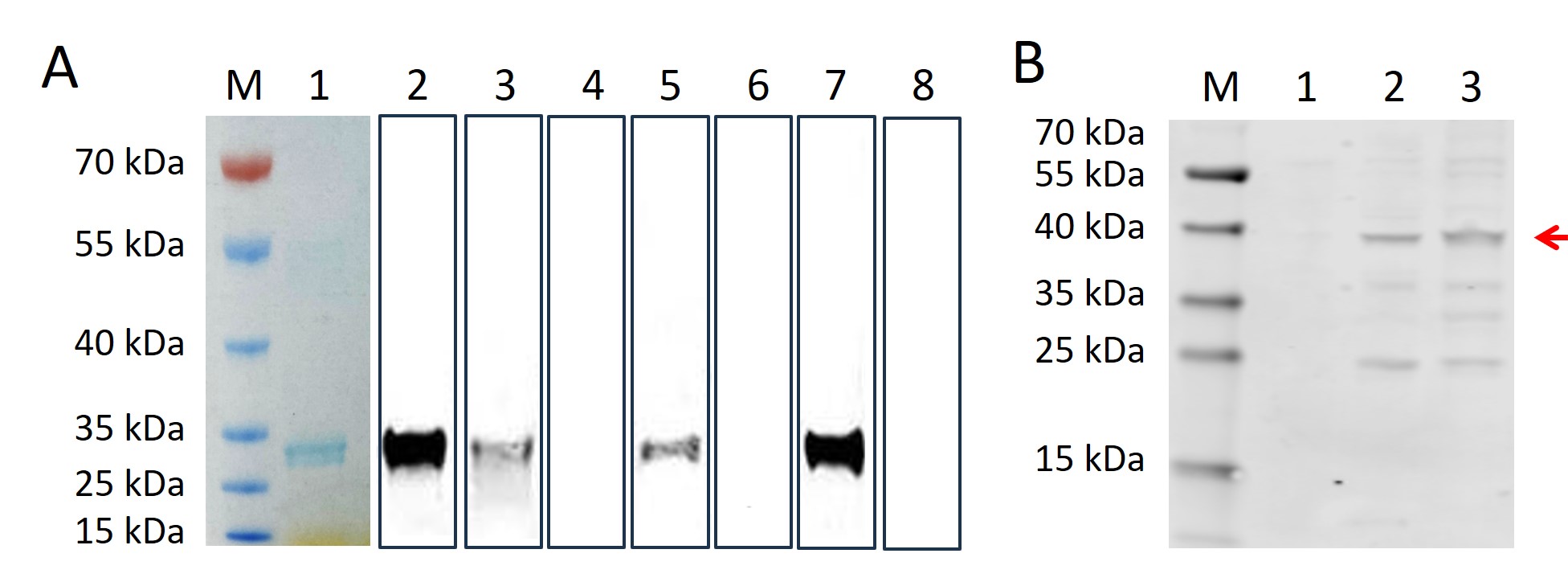

Supplement: Supplementary file 1 — Additional file 1: Fig. S1. Detection of the naïve and recombinant protein of Schistosoma japonicum erythroid Krüppel-like factor (SjEKLF). a Recombinant protein of the fragment of SjEKLF (6–214 amino acids) was resolved by 12% odium dodecyl-sulphate polyacrylamide gel electrophoresis and stained with Coomassie brilliant blue staining (Lane 1) and then detected by western blotting with an anti-His-tag mouse monoclonal antibody (Lane 2) or with patient or infected animal sera, including a mixture of serum samples (equal volumes) from 10 schistosomiasis japonica patients (Lane 3) or 10 healthy volunteers (Lane 4), a mixture of serum samples (equal volumes) from six infected BALB/c mice 42 dpi (Lane 5) or normal mice (Lane 6) and a mixture of serum samples (equal volumes) from five infected rabbits 42 dpi (Lane 7) or normal rabbits (Lane 8). b Hepatic schistosomulum (100 per well) and male adults (20 per well) were cultured in 24-well plates in RPMI 1640 for 8 h. SjEKLF in the concentrated supernatants of control group (RPMI 1640, lane 1), schistosomula group (lane 2) and male adult group (lane 3) were detected by western blotting with anti-SjEKLF antibodies. [file 13071_2023_5947_MOESM1_ESM.jpg]

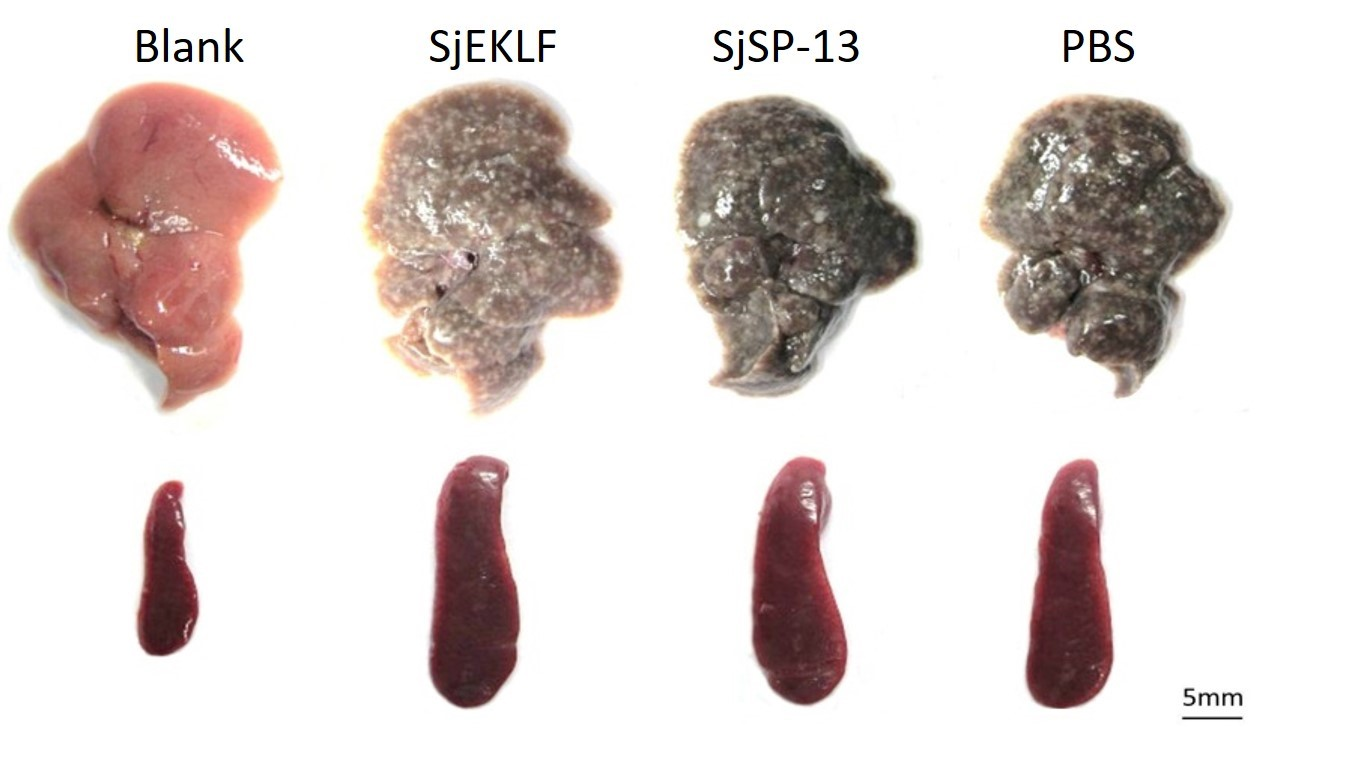

Supplement: Supplementary file 2 — Additional file 2: Fig. S2. Effect of Schistosoma japonicum erythroid Krüppel-like factor (SjEKLF) immunization on the livers and spleens of S. japonicum-infected mice. Mice were immunised with His-tagged recombinant SjEKLF, SjSP-13 (positive control) or PBS (negative control) (n = 10 per group). After immunisation, the mice were challenged with cercariae (40 ± 2 per mouse). The PBS group was immunised with PBS and did not receive cercariae. The mice were killed 42 days post infection. The morphology of the livers and spleen from each group of mice is shown. Scale bar indicates 5 mm. [file 13071_2023_5947_MOESM2_ESM.tif]
